# Pharmacological management of post-traumatic seizures in adults: current practice patterns in the UK and the Republic of Ireland

**DOI:** 10.1007/s00701-018-3683-9

**Published:** 2018-10-01

**Authors:** Harry Mee, Angelos G. Kolias, Aswin Chari, Ari Ercole, Fiona Lecky, Carole Turner, Catrin Tudur-Smith, Jonathan Coles, Fahim Anwar, Antonio Belli, Mark Manford, Timothy Ham, Catherine McMahon, Diederik Bulters, Chris Uff, John S. Duncan, Mark H. Wilson, Anthony G. Marson, Peter J. Hutchinson

**Affiliations:** 1 Department of Clinical Neurosciences, University of Cambridge & Addenbrooke’s Hospital, Cambridge, UK; 2 Surgery theme, Cambridge Clinical Trials Unit, Cambridge, UK; 3 Department of Neurosurgery, Royal London Hospital, London, UK; 4 Neurosciences Critical Care Unit and Division of Anaesthesia, University of Cambridge & Addenbrooke’s Hospital, Cambridge, UK; 5 School of Health and Related Research, University of Sheffield, Sheffield, UK; 6 Department of Biostatistics, University of Liverpool, Merseyside, Liverpool UK; 7 Department of Neurosurgery, Queen Elizabeth Hospital, Birmingham, UK; 8 Department of Neurosurgery, The Walton Centre, Liverpool, UK; 9 Department of Neurosurgery, University Hospital Southampton, Southampton, UK; 10 Department of Clinical and Experimental Epilepsy, UCL Institute of Neurology, London, UK; 11 Imperial Neurotrauma Centre, Department of Surgery & Cancer, Imperial College, London, UK; 12 Department of Molecular and Clinical Pharmacology, Institute of Translational Medicine, University of Liverpool, Liverpool, UK

**Keywords:** Post-traumatic seizures, Traumatic brain injury, Anti-epileptic medication, Seizure prophylaxis

## Abstract

**Background:**

Patient selection for seizure prophylaxis after traumatic brain injury (TBI) and duration of anti-epileptic drug treatment for patients with early post-traumatic seizures (PTS), remain plagued with uncertainty. In early 2017, a collaborative group of neurosurgeons, neurologists, neurointensive care and rehabilitation medicine physicians was formed in the UK with the aim of assessing variability in current practice and gauging the degree of uncertainty to inform the design of future studies. Here we present the results of a survey of clinicians managing patients with TBI in the UK and Ireland.

**Materials and methods:**

An online survey was developed and piloted. Following approval by the Academic Committee of the Society of British Neurological Surgeons, it was distributed via appropriate electronic mailing lists.

**Results:**

One hundred and seventeen respondents answered the questionnaire, predominantly neurosurgeons (76%) from 30 (of 32) trauma-receiving hospitals in the UK and Ireland. Fifty-three percent of respondents do not routinely use seizure prophylaxis, but 38% prescribe prophylaxis for one week. Sixty percent feel there is uncertainty regarding the use of seizure prophylaxis, and 71% would participate in further research to address this question. Sixty-two percent of respondents use levetiracetam for treatment of seizures during the acute phase, and 42% continued for a total of 3 months. Overall, 90% were uncertain about the duration of treatment for seizures, and 78% would participate in further research to address this question.

**Conclusion:**

The survey results demonstrate the variation in practice and uncertainty in both described aspects of management of patients who have suffered a TBI. The majority of respondents would want to participate in future research to help try and address this critical issue, and this shows the importance and relevance of these two clinical questions.

**Electronic supplementary material:**

The online version of this article (10.1007/s00701-018-3683-9) contains supplementary material, which is available to authorized users.

## Introduction

Traumatic brain injury (TBI) remains a significant public health problem that can result in physical, cognitive, functional and psychosocial disabilities [7].

Post-traumatic seizures (PTS) are well recognised following TBI. They are typical, albeit somewhat arbitrarily, classified as immediate (at time of impact), early (within 7 days post-TBI) or late (after 7 days) [5]. Seizures during acute hospitalisation can lead to significant derangement of brain physiology, contributing to secondary injury through energetic crisis and/or intracranial hypertension or even directly leading to brain herniation and death. Additionally, PTS during acute hospitalisation has been shown to be an independent risk factor for PTS within 12 and 24 months following TBI [9]. Late PTS can have a negative impact on quality of life, return to work, return to driving and can even result in death. The rationale for seizure prophylaxis with an anti-epileptic drug (AED) during acute hospitalisation is that the incidence of early PTS in patients following severe TBI is as high as 14% [12] and prevention of seizures can limit derangements in brain physiology, lower the risk of herniation and death and potentially prevent the development of late PTS. However, AEDs have variable positive, negative or neutral effects in both cognitive and behavioural domains [8]. They are also associated with some other side effects including bone density loss, hepatotoxicity and Stevens-Johnson Syndrome [4]. It is therefore essential to ensure that AEDs are prescribed appropriately and for the optimal duration following TBI.

Many studies of seizure prophylaxis pre-date the availability of EEG monitoring in the ITU, and in the light of evidence of the frequency of subclinical seizures in TBI, this question would benefit from re-evaluation [3]. Patients who develop PTS in the acute phase after a TBI are typically started on an AED to prevent further seizures. However, there is no high-quality evidence regarding the optimal duration of treatment for this group of patients.

There is a pressing need for high-quality evidence, and a baseline understanding of clinical practice is an essential pre-requisite for the design of an appropriate clinical trial. In 2017, a collaborative group of neurosurgeons, neurointensive care physicians and rehabilitation medicine physicians was formed with the aim of examining current practice patterns, gauging the degree of uncertainty and thus designing relevant future studies on the use of AEDs following TBI. It was agreed that a questionnaire survey would be a pragmatic way of achieving the first two objectives.

## Materials and methods

In line with the above objectives, we developed and piloted a questionnaire survey. Subsequently, the questionnaire survey was approved by the academic committee of the Society of British Neurological Surgeons (SBNS). A convenience sample of clinicians with interest in the management of patients with TBI and/or seizures was asked to complete the survey. A secure online survey tool was used to disseminate the questionnaires via the electronic mailing lists of the SBNS, British Neurosurgical Trainees Association (BNTA) and included in the Association of British Neurologists newsletter. The survey was also promoted by the Twitter accounts of the SBNS (@The_SBNS), BNTA (@e1v1m1), British Neurotrauma Group (@bntg_uk), British Neurosurgical Trainee Research Collaborative (@BNTRC) and Association of British Neurologists (@theABN_Info).

Our target audience were clinicians who were involved with the acute and long-term management of TBI patients, who were linked with adult trauma-receiving neurosurgical units. We disseminated the survey to neurousurgeons, intensive care medicine/anaesthesia, neurology, emergency medicine and rehabilitation medicine. Due to the wide dissemination of the questionnaire through social media platforms, calculation of the response rate is not possible; 95% confidence intervals have been used and documented as (%-%) after the figures.

## Results

The online questionnaire was completed by 117 clinicians from a range of specialties, but predominately neurosurgeon-neurosurgery (*n* = 89, 76%), intensive care medicine/anaesthesia (*n* = 24, 21%), neurology (*n* = 2, 2%), emergency medicine and rehabilitation medicine (*n* = 1 each). The majority of the respondents were consultants (*n* = 78, 67%), while the remaining were trainees or fellows (*n* = 39, 33%). There were respondents from 30 of the adult trauma-receiving neurosurgical units, 29 in the UK and 1 from Ireland. There was at least 1 response from a Consultant from 21/30 (66%) of the adult receiving neurosurgical units. The questionnaire disseminated can be found in the online supplementary material.

### Seizure prophylaxis

**Fig. 1:**
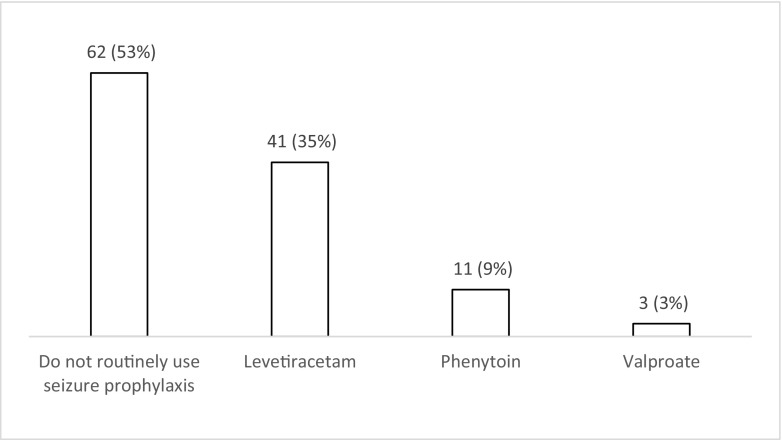
Responses to question: ‘Which anti-epileptic drug do you use as first choice for seizure prevention (i.e. the patient has not had a seizure) in moderate to severe traumatic brain injury during the acute phase?’

**Fig. 2:**
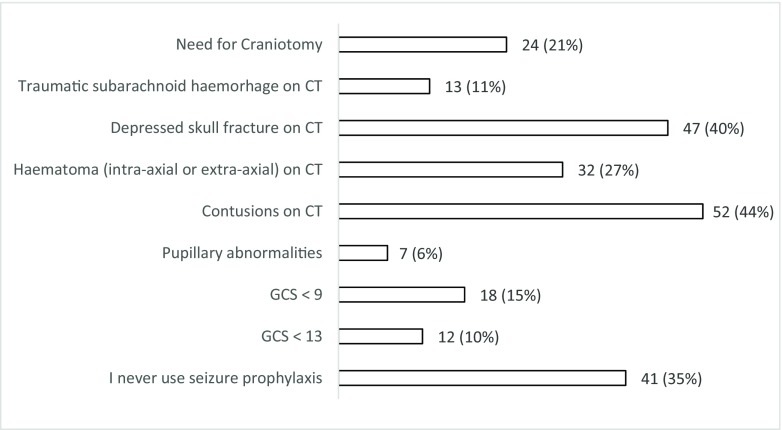
Responses to question: ‘Which factors influence your decision to start seizure prophylaxis?’ (can select more than one answer)

**Fig. 3:**
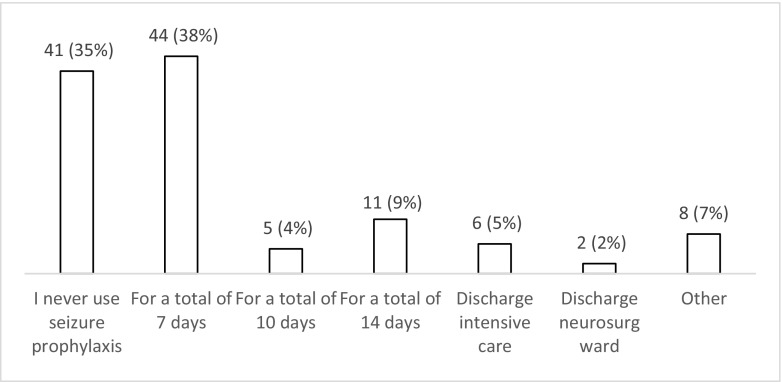
Responses to question: ‘If you start seizure prophylaxis during the acute phase, how long do you continue (assuming that no seizures occur)?’

**Fig. 4:**
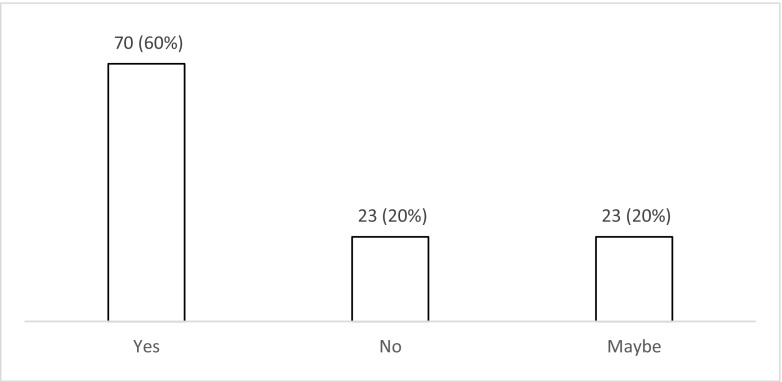
Responses to question: ‘Do you think that there is uncertainty/equipoise about the use (or not) of seizure prophylaxis in moderate to severe traumatic brain injury during the acute phase?’

**Fig. 5:**
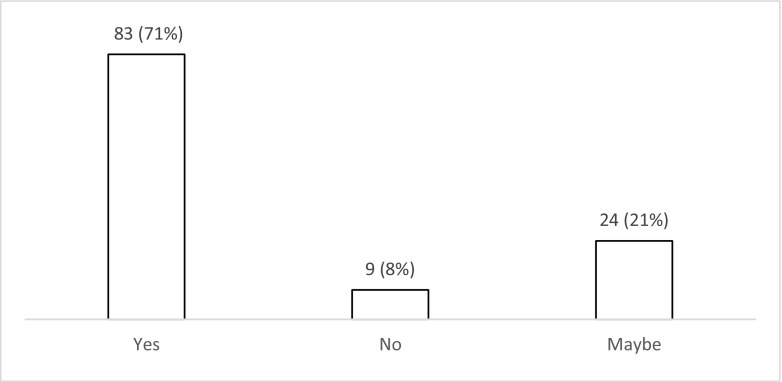
Responses to question: ‘Would you participate in a randomised trial to address seizure prophylaxis in moderate to severe traumatic brain injury during the acute phase?’

Fifty-three percent (*n* = 62; 44–62%) of respondents do not use seizure prophylaxis routinely compared to 47% (*n* = 55; 31–50%) who do so for patients with a moderate or severe TBI during the acute phase (Fig. 1). Of those who use prophylaxis, 75% (*n* = 41/55; 61–85%) chose levetiracetam over phenytoin (*n* = 11/55; 20% (11–33%)) or valproate (*n* = 3/55; 5% (1–16%). When asked about factors influencing their decision to start prophylaxis (Fig. 2), 65% of the respondents (*n* = 76; 55–73%) selected at least one factor. The top five factors influencing the decision to start seizure prophylaxis are the presence of contusions on CT (*n* = 52), depressed skull fracture (*n* = 47), intra-axial or extra-axial haematoma on CT (*n* = 32), need for craniotomy (*n* = 24) and a GCS < 9 (*n* = 18). When asked about the length of prophylaxis, the majority (*n =* 44; 58% (29–47%)) prescribe prophylaxis for 7 days (Fig. 3). Finally, the majority (*n* = 70; 60% (50–69%)) felt that there is uncertainty/equipoise surrounding the use of seizure prophylaxis (Fig. 4) with 71% (*n* = 83; 62–79%) stating that they would participate in a randomised trial to address seizure prophylaxis in moderate to severe TBI during the acute phase (Fig. 5).

### Treatment of early PTS

**Fig. 6:**
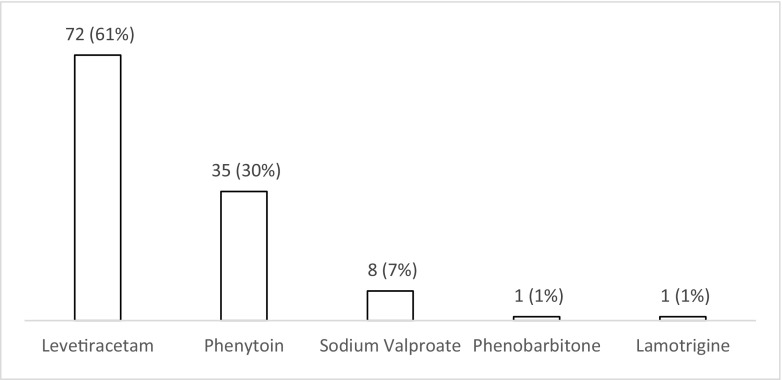
Responses to question: ‘Which anti-epileptic drug do you use for a patient with traumatic brain injury who has had seizure(s) during the acute phase?’

**Fig. 7:**
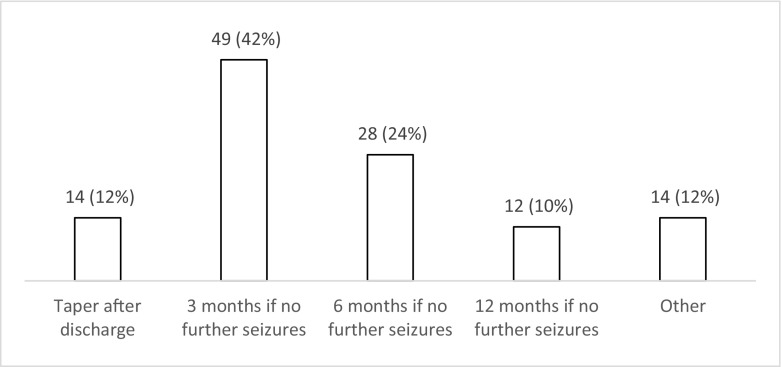
Responses to question: ‘If you initiate treatment with anti-epileptics for seizures in the acute phase after traumatic brain injury, how long do you continue for?’

**Fig. 8:**
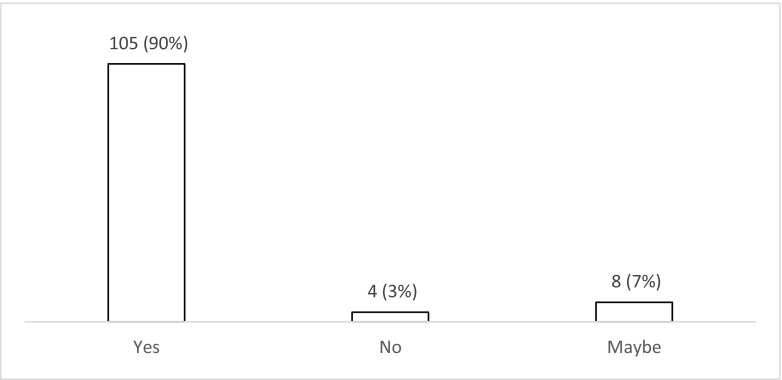
Responses to question: ‘Do you think that there is uncertainty about the duration of treatment with anti-epileptic drugs for seizures occurring in the acute phase after traumatic brain injury?’

**Fig. 9:**
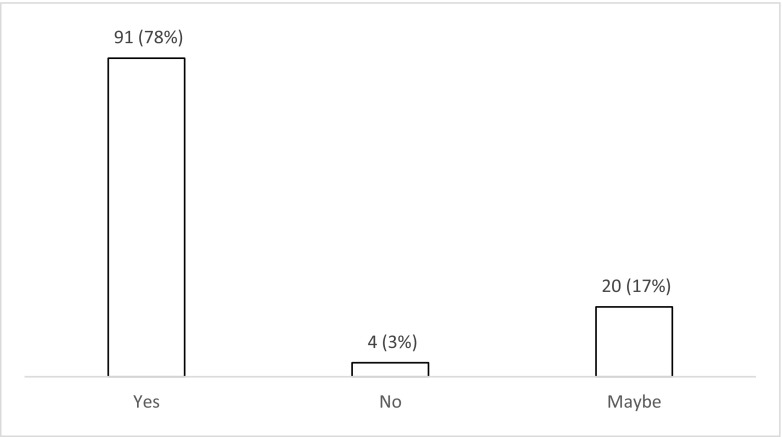
Responses to question: ‘Would you participate in a randomised trial to address duration of treatment with anti-epileptic drugs for seizures occurring in the acute phase after traumatic brain injury?’

The majority of respondents (*n* = 72; 62% (52–70%)) use levetiracetam for patients with PTS during the acute phase (Fig. 6). Nearly one third (*n* = 35; 30% (22–39%)) use phenytoin with valproate favoured by less than 10% (*n* = 8; 3–13%). There was variation in the duration of treatment with AEDs (Fig. 7), with 42% (*n* = 49; 33–51%) continuing treatment for 3 months if no further seizures occur, 24% (*n* = 28; 17–33%) for 6 months, 10% (*n* = 12; 5–18%) for 12 months and 12% (*n* = 14; 7–20%) tapering after discharge from the hospital (Fig. 7). Ninety percent (*n* = 105; 82–94%) stated that there is uncertainty regarding the optimal duration of treatment with AEDs for PTS occurring during acute hospitalisation (Fig. 8), with 78% (*n* = 91; 69–85%) stating that they would participate in a randomised trial to address duration of treatment (Fig. 9).

**Fig. 10:**
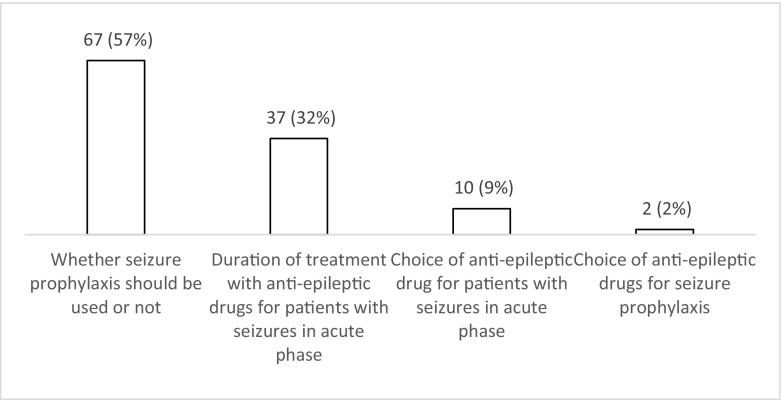
Responses to question: ‘There are a few uncertainties in the management of post-traumatic seizures that could be addressed by future studies. Which one is the most important priority in your opinion?’

When respondents were asked to select the top priority for future research in the field of PTS, 57% (*n* = 67; 48–66%) answered seizure prophylaxis, nearly one third (*n* = 37; 32% (24–41%)) duration of treatment for PTS during the acute phase, and 10% favoured research on the type of AEDs that should be used (Fig. 10).

## Discussion

The survey findings confirm that there is significant variation in the practice across the UK and Ireland with regard to the use of seizure prophylaxis and the duration of treatment with AEDs after early PTS. A Cochrane review [14], concluded that there is ‘low-quality evidence that early treatment with an AED compared with placebo or standard care reduced the risk of early post-traumatic seizures’ and that ‘there was no evidence to support a reduction in the risk of late seizures or mortality’. Despite that, nearly half of the respondents routinely use prophylactic AEDs (47%). The 2016 ‘Brain Trauma Foundation’ guidelines [1] stated that ‘phenytoin is recommended to decrease the incidence of early PTS, when the overall benefit is felt to outweigh the complications associated with such treatment’, but concluded that ‘there was insufficient evidence to support a Level I recommendation for the topic of post-traumatic seizures’ and are calling for further trials.

The survey showed that the two most commonly used AEDs, for prophylaxis or treatment, are levetiracetam and phenytoin with the former having surpassed the latter in popularity. This reflects the findings of a recent survey of US clinicians [10], which showed that 74% of the respondents prefer levetiracetam for seizure prophylaxis, with only 10% favouring phenytoin. A similar trend has also recently been demonstrated in Europe [6].

Temkin et al. [12] demonstrated that phenytoin given for 1-year versus placebo decreased early PTS (within 7 days) from 14.2% down to 3.6%, but seizure rate did not vary after 7 days. Therefore, the available evidence, so far, suggests prophylaxis treatment is beneficial for reduction of early PTS only. Our study shows a variable prophylaxis rate, with 52% not using prophylaxis routinely and 60% being uncertain about the use of prophylaxis. A prospective, randomised, single-blinded study by Szaflarski et al. [11] showed no difference between the seizure rates of phenytoin or levetiracetam. However, this was a small study, and it is noted that further exploration is required. Due to its superior side effect profile and the fact, there is no need for plasma monitoring levetiracetam has become the AED of choice, with 58% of respondents choosing to use this drug.

PTS during and after acute hospitalisation are often harmful. Recurrent PTS post-TBI can negatively impact on quality of life, return to work/driving and can even lead to death. PTS during acute hospitalisation has been shown to be an independent risk factor for PTS within 12 and 24 months following TBI [9]. AEDs are the mainstay of treatment for patients with PTS but are associated with side effects that, if serious, can negatively impact on quality of life, cognition and general health [4, 8]. Patients with acute PTS are typically started on an AED to prevent seizure recurrence. The optimal duration of treatment remains unclear [13] but as TBI carries an increased risk of epilepsy as a consequence of recurrent seizures [2], further trials are necessary to try and answer these important questions.

Although we acknowledge there are limitations in questionnaire surveys and appreciate that the response rate of online surveys is not possible to know due to the multiple channels of dissemination, we feel that having over 100 responses from the majority of adult trauma-receiving neurosurgical units in the UK and Ireland provides a reasonable overview of the current practice patterns. A further limitation is the fact that there were only Consultant responses from two thirds of the units; however, trainees and speciality doctors in these units play an active role in the management of TBI patients and PTS and therefore the value of having their views cannot be ignored and commonly will reflect the views of the consultants.

The survey results demonstrate that there is significant uncertainty as to the duration of treatment of acute PTS, and also, uncertainty surrounding whether prophylaxis for PTS should be given. The results of the survey are not surprising as they underline the known uncertainity of current practices across the UK and Ireland and confirms the need for future research around this topic.

The uncertainties are most likely due to the lack of high-quality data investigating the duration of treatment and prophylaxis of PTS. The fact that the majority of the respondents are willing to collaborate on future studies highlights the importance of this subject to the community of clinicians caring for TBI patients in the UK.

## Conclusions

The current paper demonstrates the variation in practice and uncertainty in both described aspects of the management of patients with TBI. The majority of respondents would want to participate in future research to help try and address these issues, and this shows the importance and relevance of these two clinical questions. Ultimately, class I evidence is necessary to provide clinicians with a better evidence base and achieve further improvements in the outcome of patients with TBI and PTS.

## Electronic supplementary material

ESM 1 (DOCX 19 kb)
