# Supplementary material for: Pharmacological management of post-traumatic seizures in adults: current practice patterns in the UK and the Republic of Ireland
Source: Acta Neurochir (Wien). 2018 Oct 1;161(3):457–64. doi: 10.1007/s00701-018-3683-9 (PMC6407744; doi:10.1007/s00701-018-3683-9)
Supplement: Supplementary file 1 — (DOCX 19 kb) [file 701_2018_3683_MOESM1_ESM.docx]

**Management of post-traumatic seizures in adults**

Dear Colleagues,

This survey seeks your opinion on the management of seizures in adult patients with traumatic brain injury. As you are aware, there is some variation in terms of seizure prophylaxis, agents used, and duration of treatment; the current survey aims to quantify this variation.

Thank you for taking the time to complete this survey.

Please tick the appropriate responses.

**Section 1: General information**

1. What is your speciality?
   1. Intensive Care Medicine
   2. Neurology
   3. Neurosurgery
   4. Rehabilitation Medicine
   5. Other…………………………………………………………………
2. What is your grade?
   1. Consultant
   2. Trainee
3. Which hospital are you working in?
   1. Location …………………………………………………………………

**Section 2: Seizure Prophylaxis**

1. Which anti-epileptic drug do you use as first choice for seizure prevention (i.e. the patient has not had a seizure) in moderate to severe traumatic brain injury during the acute phase?
   1. I do not routinely use seizure prophylaxis
   2. Levetiracetam
   3. Phenytoin
   4. Valproate
   5. Other …………………………………………………………………
2. Which factors influence your decision to start seizure prophylaxis (you can select > 1 factors)?
   1. I never use seizure prophylaxis
   2. GCS < 13
   3. GCS < 9
   4. Pupillary abnormalities
   5. Contusions on CT
   6. Haematoma (intra-axial or extra-axial) on CT
   7. Depressed skull fracture on CT
   8. Traumatic subarachnoid haemorrhage on CT
   9. Need for craniotomy
   10. Other …………………………………………………………………
3. If you start seizure prophylaxis during the acute phase, how long do you continue (assuming that no seizures occur)?
   1. I never use seizure prophylaxis
   2. For a total of 7 days
   3. For a total of 10 days
   4. For a total of 14 days
   5. Until discharge from intensive care
   6. Until discharge from neurosurgical ward
   7. Other …………………………………………………………………
4. Do you think that there is uncertainty / equipoise about the use (or not) of seizure prophylaxis in moderate to severe traumatic brain injury during the acute phase?
   1. Yes
   2. No
   3. Maybe
   4. Any further comments …………………………………………………………………
5. Would you participate in a randomised trial to address seizure prophylaxis in moderate to severe traumatic brain injury during the acute phase?
   1. Yes
   2. No
   3. Maybe

**Section 3: Management of early post-traumatic seizures**

1. Which anti-epileptic drug do you use for a patient with traumatic brain injury who has had seizure(s) during the acute phase?
   1. Carbamazepine
   2. Lamotrigine
   3. Levetiracetam
   4. Phenytoin
   5. Valproate
   6. Other …………………………………………………………………
2. If you initiate treatment with anti-epileptics for seizures in the acute phase after traumatic brain injury, how long do you continue for?
   1. I start tapering after discharge from the hospital
   2. For a total of 3 months if no further seizures have occurred
   3. For a total of 6 months if no further seizures have occurred
   4. Other …………………………………………………………………
3. Do you think that there is uncertainty about the duration of treatment with anti-epileptic drugs for seizures occurring in the acute phase after traumatic brain injury?
   1. Yes
   2. No
   3. Maybe
   4. Any further comments …………………………………………………………………
4. Would you participate in a randomised trial to address duration of anti-convulsant treatment in moderate to severe traumatic brain injury?
   1. Yes
   2. No
   3. Maybe

**Section 4: Further Research**

1. There are a few uncertainties in the management of post-traumatic seizures that could be addressed by future studies. Which one is the most important priority in your opinion?
   1. Whether seizure prophylaxis should be used or not
   2. Choice of anti-epileptic drugs for seizure prophylaxis
   3. Duration of treatment with anti-epileptic drugs for patients with seizures in the acute phase
   4. Choice of anti-epileptic drugs for patients with seizures in the acute phase
   5. Other…………………………………………………………………
2. If you have any further comments, please specify below

Thank you for your time. If you are interested in collaborating in future studies, please leave your email address

……………………………………………………………………………………………………………………………………………...
